# Study on material and mechanical characteristics of silicone rubber shed of field-aged 110 kV composite insulators

**DOI:** 10.1038/s41598-023-35701-8

**Published:** 2023-10-06

**Authors:** Lin Mu, Bo Wang, Jinpeng Hao, Ziyi Fang, Yu Wang

**Affiliations:** 1 School of Electrical and Electronic Information, Xihua University, No. 999, Jinzhou Road, Jinniu District, Chengdu, China; 2 State Grid Ningxia Electric Power Company Electric Power Research Institute, No. 716, Huanghe East Road, Yinchuan, China; 3 School of Electrical and Automation, Wuhan University, No. 299, Bayi Road, Wuhan, China

**Keywords:** Electrical and electronic engineering, Materials chemistry

## Abstract

Composite insulators have excellent performance and are more and more widely used in power grid. The performance of composite insulators with different service duration will decline in varying degrees, which could pose a threat to the safe operation of power grid. In order to investigate the influence of service duration and electric field strength on insulator shed performance, the sheds at different positions of insulators with different service duration are sampled. The hydrophobicity, material and mechanical properties of the samples are tested, and then the micro material properties tests are performed in terms of SEM, FTIR and XPS tests. Based on the above test results, the aging law and its mechanism of silicone rubber sheds are analyzed. The results reveal that the performance of insulator shed gradually decline with the increase of service life. The hydrophobicity and hardness of high-voltage end insulator are similar to that of middle section insulator, while other parameters are obviously different, indicating that the electric field can aggravate the aging. FTIR results show that the main chain and hydrophobic side chain of silicone rubber are destroyed, and the oxygen-containing groups increased, indicating that thermal oxygen aging occurred during operation. XPS and SEM results show that the crosslinking degree of silicone rubber increases and the porosity increases. The above changes in the microstructure of silicone rubber lead to the decline of insulator performance.

## Introduction

Due to the development of urbanization and industrialization, air pollution is increasingly serious. After the surface of outdoor insulator is polluted, the insulation performance will decrease greatly in humid weather, which threatens the safe and stable operation of transmission lines. In the past two decades, the frequency of pollution flashover on transmission lines in China has increased dozens of times^1,2^.

Composite insulators mainly consist of umbrella sheds, metal clamps, a glass fiber rod and rod housing. The shed is made of high temperature vulcanized (HTV) silicone rubber (SIR), which plays a major role in preventing pollution flashover. Because of its good hydrophobicity and hydrophobicity migration, the composite insulator has better pollution flashover performance than porcelain insulator and glass insulator. Composite insulators have been used in China since 1980s. At present, there are more than two million composite insulators in operation^3,4^. Composite insulator shed is made of HTV, which will be aged gradually during operation, resulting in the decrease of hydrophobicity, hardening, cracking and other phenomena^5–7^. The insulation performance of insulators with different service duration will be reduced to various degrees, which may cause failure risks to the operation of transmission lines. Therefore, the research on the aging characteristics of insulators is of great significance to improve the safety level of the lines.

The aging characteristics of composite insulator shed are closely related to its operation performance, which has attracted the attention of many experts and scholars. Many studies employ static contact angle test method to analyze the aging characteristics of SIR samples^8–11^. The relationship between hardening, cracking and aging degree of composite insulator sheds has also been studied^12–14^. The sheds of composite insulator samples from different regions are collected and tested by Chen. It is found that there is a correlation between the degree of crosslinking and hardness of the SIR material^15^. In reference^16^, the cracking characteristics of composite insulator sheath are observed by microscope from different angles. There are also many studies on the aging rule of SIR from the perspective of electrical and material properties^17–20^. The polymer structure of silicone rubber corona aged under different humidity was measured through nuclear magnetic resonance and Fourier transform infrared spectroscopy. The result shows that the aging degree will increase with the increase of relative humidity^21^. The electrical and material parameters of shed samples at different positions of composite insulator strings are tested by Rowland. The results show that the parts with strong sunlight and sea breeze is aged more severely. The aging of silicone rubber is mainly oxidation process, which leads to the increase of Si-OH group content. The wet flashover voltage of samples with serious aging is lower^22^. The surface conductivity and trap density/energy level of corona aged and natural aged SIR samples are measured by Song. The results show that the conductivity and trap density increase with the increase of aging duration^23^.

In the past two years, relevant literature has mainly focused on the impact of corona discharge, environmental humidity, haze, and end heating on the composite insulator umbrella shed. The characteristics of the umbrella shed studied mainly include its hydrophobicity, appearance, microscopic morphology, and functional groups^24–27^. There is little research on the variation law of viscoelasticity and tear strength of field-aged composite insulator shed at present. The study on aging characteristics of insulators with different service duration and sheds at different positions of insulator still needs to be supplemented and improved.

In this paper, the shed samples of composite insulator with different operating years are collected. Through the appearance, hydrophobicity, density, hardness, tear strength and viscoelasticity tests, the change rules of mechanical strength and macro material properties of SIR during field aging are analyzed. The aging mechanism of SIR is analyzed combined with microstructure obtained by scanning electron microscopy (SEM), attenuated total reflection Fourier transform infrared spectrometer (ATR-FTIR) and X-ray photoelectron spectroscopy (XPS).

### Insulator samples

The insulator samples of different service duration are taken from the 110 kV transmission line in northern China. The model of the insulators is FXBW-110/100, which is of large and small shed structure. There are 16 large sheds for this type of insulator and the large sheds at the middle section and lower end is the main research object. The main pollution sources are dust from agriculture and traffic. The pollution level of insulator with 20 years operation is heavy, and that of the other operated insulators is medium, referring to the standard IEC 60815^28^. Sample information is shown in the Table 1.

**Table 1:** Sample information.

Number	Service duration/year	Rated voltage /kV	Pollution level	Amount
1	0	110	–	1
2	10	110	c	2
3	20	110	d	2
4	26	110	c	2

## Experimental setup

### Appearance and hydrophobicity

The sheds at the middle section and lower end (high voltage end) of the insulator are tested to analyze the influence of electric field strength on aging.

The appearance of insulator shed is visually inspected, and then the hydrophobicity is tested by Hydrophobicity Classification (HC) method. According to the shape of water droplets and the percentage of wetted surface, the hydrophobicity classification level can be divided into HC1–HC7. HC1 corresponds to the fully hydrophobic surface and HC7 corresponds to the fully hydrophilic surface^29^.

The edge area of the large umbrella shed are taken and cut into 40 mm × 20 mm square samples. In order to compare the effect of pollution on hydrophobicity, some samples are cleaned with absolute ethanol and deionized water, while others are not treated.

A contact angle meter (model SL150E) is employed to quantitatively evaluate the hydrophobicity of samples. The volume of the water drop is set to 4 μL and the sampling frequency of the camera is set to 2 Hz. After dripping the water droplets on the surface of the specimen for 5 s, the camera collects pictures of water drops. The static contact angle is calculated based on θ/2 method. 6 different points on one sample are tested and the average value of the result are taken as the contact angle of the sample.

### Hydrophobicity loss and recovery

In case of rain, the hydrophobicity of the shed may be lost. Then the small siloxane molecules inside the silicone rubber shed will migrate outward, gradually restoring the hydrophobicity of the surface. Hydrophobic migration is related to the organic component content of silicone rubber, which reflects the aging degree of umbrella shed. Therefore, the loss and recovery test of hydrophobicity of silicone rubber of shed is carried out.

The cleaned samples are soaked in deionized water for 96 h in the laboratory environment (temperature 20 ± 5℃, relative humidity 50–70%). The soaked samples are wiped dry with filter paper, and then the static contact angle are measured 10 min later. After the hydrophobicity loss measurement, the samples are placed in the laboratory environment for 48 h, during which the static contact angle is measured at regular intervals to obtain the hydrophobicity recovery law.

### Density, hardness and tear strength

The square specimens are cleaned and weighed with an electronic balance of 0.1 mg accuracy. The beaker containing water is put on the balance with indication set to zero, then the specimen hang with wire is immersed in water to obtain the mass of water equal to the volume of the specimen. The density of the specimen can be obtained through the following formula.

1 $$\rho_{{\text{s}}} = \frac{{\rho_{{\text{w}}} m_{{\text{s}}} }}{{m_{{\text{s}}} - m_{{\text{w}}} }}.$$

Among them, *ρ*_w_ and *m*_w_ represents the density and mass of water. *ρ*_s_ and *m*_s_ represents the density and mass of SIR.

The hardness of 6 positions on the surface of the sheds are measured by Shore hardness tester. The average value of the obtained data is taken as the hardness value of the sample.

The shed is processed into 2 mm thick rectangular specimen by a planer and a mould. The specimen is stretched at a constant speed of 450 mm/min by a tensile machine to measure its tear strength.

### Viscoelasticity

Viscoelasticity refers to the viscous and elastic properties of high molecular polymers such as SIR under stress, which is related to hardness and microstructure. Dynamic thermo mechanical analysis (DMA) is a new method for measuring viscoelasticity of materials.

DMA measures the relationship between dynamic modulus and temperature by applying periodic stress to the sample. Assuming that a force with an amplitude of *F*a is applied to the sample, the displacement amplitude *L*a of the sample and the phase difference δ between the force and the displacement can be measured. The dynamic modulus can be calculated by the following formula.

2 $$E^{*} = \frac{{F_{a} }}{{L_{a} }} \times g,$$

where E^*^ is the composite modulus and *g* is the geometry factor.

3 $$\begin{array}{*{20}l} {E^{\prime\prime} = E^{*} \times \sin \delta } \\ {E^{\prime} = E^{*} \times \cos \delta } \\ {\tan \delta = \frac{{E^{\prime\prime}}}{{E^{\prime}}}} \\ \end{array} ,$$

where *E*ʹʹ represents the loss modulus, which is directly proportional to the converted heat energy, *E*ʹ represents the storage modulus, which is directly proportional to the stored elastic energy, and tanδ represents the loss factor, which can characterize the relative viscoelastic strength of the material. Square specimens are cleaned and dried for DMA test. The equipment model is DMS710 and the load mode of the test is compression. The frequency of sinusoidal stress is set to 1 Hz and the temperature range is set to room temperature to 250 °C at a heating rate of 3 °C/min.

### Micro performance analysis

In order to analyze the micro morphology of the aged shed and its relationship with mechanical properties, the cross section after the shed tear test is observed by SEM (model Quanta 200). The magnification is set to 1000 times.

The silicone rubber from the upper surface of the umbrella shed edge are cleaned and dried for ATR-FTIR (model Nicolet 5700) detection. The functional groups in the specimen are in vibration state and will absorb infrared light of specific frequency. The measured spectra can reveal the attenuation of infrared light at each characteristic frequency, so as to reflect the content of characteristic functional groups in the sample. The measured depth range is 1–10 μm. The infrared spectrum range is set to 400–4000 cm^−1^ with an accuracy of 0.5 cm^−1^.

The same samples tested by FTIR are used for XPS detection. The equipment model of XPS is Escalab250xi. The penetration depth of X-ray is 1–10 nm, so the element content and atomic valence state in the depth range of 10 nm on the sample surface can be analyzed.

## Results and discussion

### Appearance and hydrophobicity

**Figure 1:**
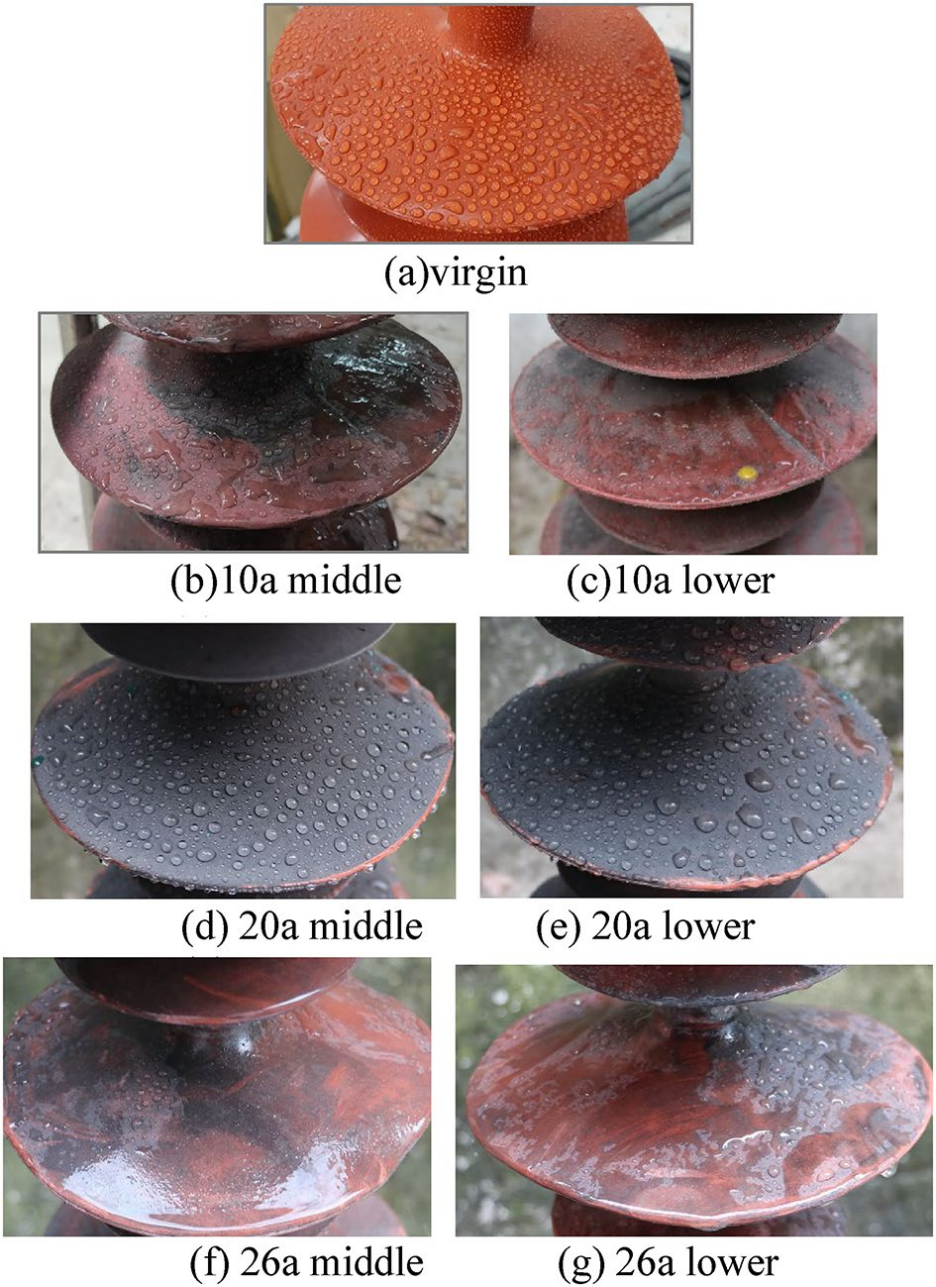
Appearance of insulator shed after water spraying.

The insulator shed after water spraying is shown in the Fig. 1.

It can be seen that the color of the shed of the insulator after operation is obviously lighter, which is a sign of aging. The sheds have different degrees of hardening, but there is no cracking. The hydrophobicity grade of the virgin sample is HC1. The hydrophobicity grades of 10a, 20a and 26a samples are HC3–5, HC1–2 and HC5–7 respectively. The hydrophobicity of polluted insulators after service is worse than that of the virgin sample. The hydrophobicity of 20a insulator is better than that of 10a insulator, which is mainly because the pollution layer attached to the sample surface is thicker, and more hydrophobic substances migrate and adsorb in it^30^. The pollution distribution of one umbrella shed of 10a and 26a samples is uneven, so the variation range of hydrophobicity is also large. There is little difference in hydrophobicity between the lower end and the middle section of the same insulator.

The hydrophobicity of the clean and polluted surfaces of the specimen is expressed by the static contact angle and the results are shown in the Table 2.

**Table 2:** Hydrophobicity of clean and polluted surface.

Specimen	Polluted surface/°	Clean surface/°	Difference/°
0a	118.87	118.87	0
10a middle	97.66	115.85	18.19
10a lower	102.68	116.92	14.24
20a middle	126.49	115.78	− 10.71
20a lower	126.67	119.03	− 7.64
26a middle	95.04	112.07	17.03
26a lower	99.31	115.54	16.23

The contact angle of the shed after operation is more than 90°, indicating that it still maintains hydrophobicity. The static contact angle of polluted surface of 20a sample is 126°, which is better than that of the virgin sample. After removing the pollution, the static contact angle is reduced by 10°. The main reason is that a large number of low molecular weight (LMW) substances produced by the scission of SIR molecular chain during aging migrate outward^31,32^. As the 20a sample is heavily polluted, the pollution plays the role of preserving LMW, which increases the surface free energy of the pollution layer and the cleaned shed. The surface of the pollution layer with greater roughness also has a retention effect on the water droplets, which further increases the apparent contact angle. The pollution on the 10a and 26a sample surface may often be removed due to wind and rain scouring, resulting in less LMW in the pollution layer. Therefore, the hydrophobicity of the polluted surface is worse than that of the shed surface. According to the contact angle of 0a, 10a and 26a samples, the hydrophobicity gradually decreases with the growth of operation years. The hydrophobicity of the shed of the lower end is slightly better than that of the middle section, indicating that the hydrophobicity may not be weakened by the electric field.

### Hydrophobicity loss and recovery

**Figure 2:**
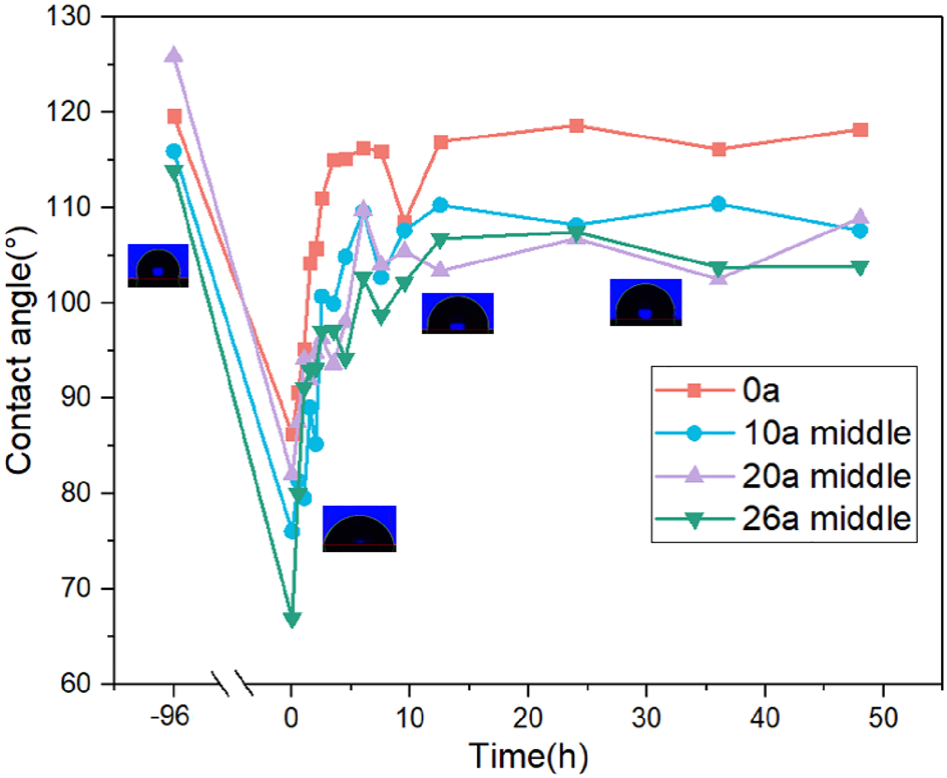
Hydrophobic loss and recovery characteristics.

According to the LMW migration theory, the hydrophobic LMW in the SIR bulk will diffuse to the surface driven by the concentration difference^33,34^. Therefore, the speed of hydrophobicity loss and recovery is related to the content of organic components in silicone rubber. The change trend of contact angle during the loss and recovery of hydrophobicity is shown in the Fig. 2.

It can be seen that an apparent decline of contact angle appears for the samples after 96 h immersion. The hydrophobicity of the virgin sample is the best, and the contact angle is still 85°. The 26a sample with longest service duration has the worst hydrophobicity, and the contact angle is only 65°. After being taken out of water, the contact angle of the sample increases rapidly and reaches saturation at 10 h with the diffusion of LMW to the surface. After 48 h of recovery, the contact angle of the virgin sample basically returns to the level before the immersion test. The contact angle of the sample after operation is still 7°–16° lower than that before immersion test. The steady state contact angle shows a negative correlation with the service duration indicating that the insulator shed gradually ages and its organic component content decreases with the increase of service duration.

### Density, hardness and tear strength

**Figure 3:**
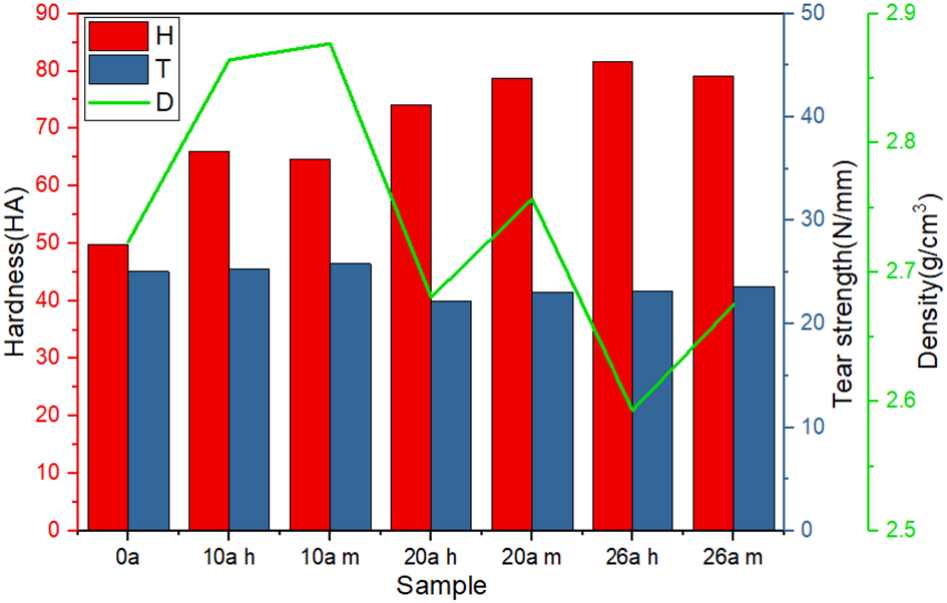
The density, hardness and tear strength of samples.

After the hardness and tear strength are reduced, the shed is easy to crack under the action of external force, resulting in the decrease of insulation strength of insulator. Density, hardness and tear strength are also closely related to microstructure of the silicone rubber. The Fig. 3 presents the density, hardness and tear strength of each sample.

It can be seen that the hardness of the shed increases gradually with the increase of service duration. The polydimethylsiloxane (PDMS) in SIR may undergo rearrangement degradation process and produce a large amount of cross-linking networks resulting in poor molecular flexibility and an increase in macro hardness.

The tear strength first increases and then decreases. The main reason is that the increase of crosslinking degree first increases the tear strength. When the crosslinking point increases further, the SIR becomes brittle. Meanwhile, the crosslinking agent gradually decomposes and the reinforcing filler such as white carbon black precipitates, which makes the crosslinking network uneven resulting in the decrease of tear strength. By comparing the measurement results of several samples, it is found that the tear strength of the shed at the lower end is lower than that at the middle section, indicating that the high electric field strength aggravates the deterioration of the mechanical properties of insulator shed.

The density shows the consistent trend as the tear strength. Relevant studies show that the density of SIR is negatively correlated with the content of base rubber^35^. Therefore, it can be inferred that during operation, the oxidation reaction on the shed surface leads to the decrease of base rubber content and the increase of density. Then, due to the precipitation of fillers, the shed density decreases.

### Viscoelasticity

**Figure 4:**
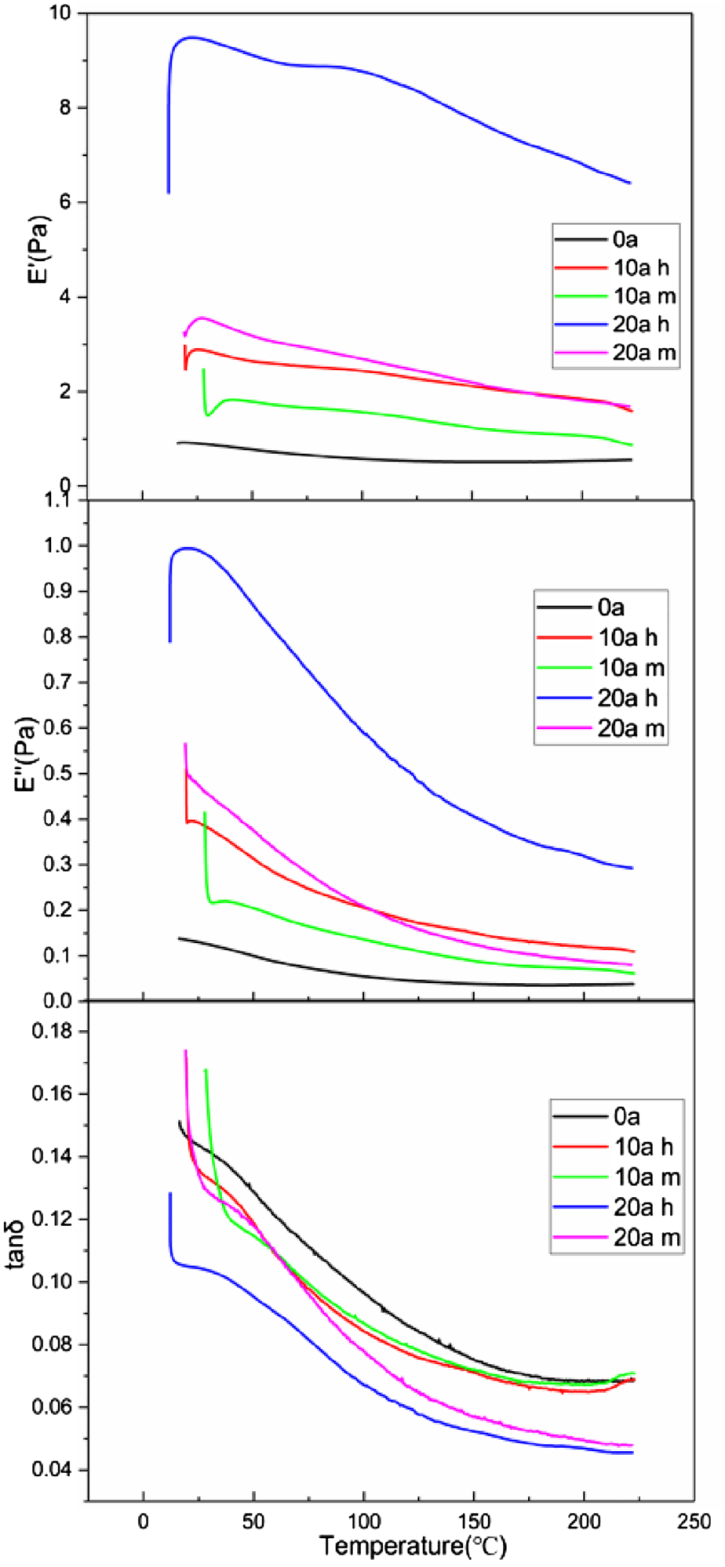
The variation curve of viscoelastic parameters of the sample.

The temperature characteristic curve of viscoelastic parameters of the sample is shown in the Fig. 4.

It can be seen that the loss factor shows a downward trend indicating that the test temperature is higher than the glass transition temperature and the samples are in high elastic state. In the long term operation, the insulator shed has gone through the aging process of fracture rearrangement. The increase of cross-linking point in the molecule leads to the enhancement of the rigidity of silicone rubber. Therefore, the storage modulus increases with the increase of service duration. The formation of SIR internal cross-linking network will increase the internal friction, so the loss modulus also increases with the service duration. The storage modulus and loss modulus of the lower end shed are greater than that of the middle section shed for the same insulator, and the longer the service duration, the greater the difference between them, indicating that the electric field may aggravate the deterioration of viscoelasticity of silicone rubber. The longer the service duration, the more significant the decrease of viscoelastic modulus with the increase of temperature. The main reason is that the increase of temperature strengthens the movement of molecules, thus destroying the chemical bonds between molecular chains. Therefore, the samples with higher crosslinking degree are more affected by temperature.

### SEM

**Figure 5:**
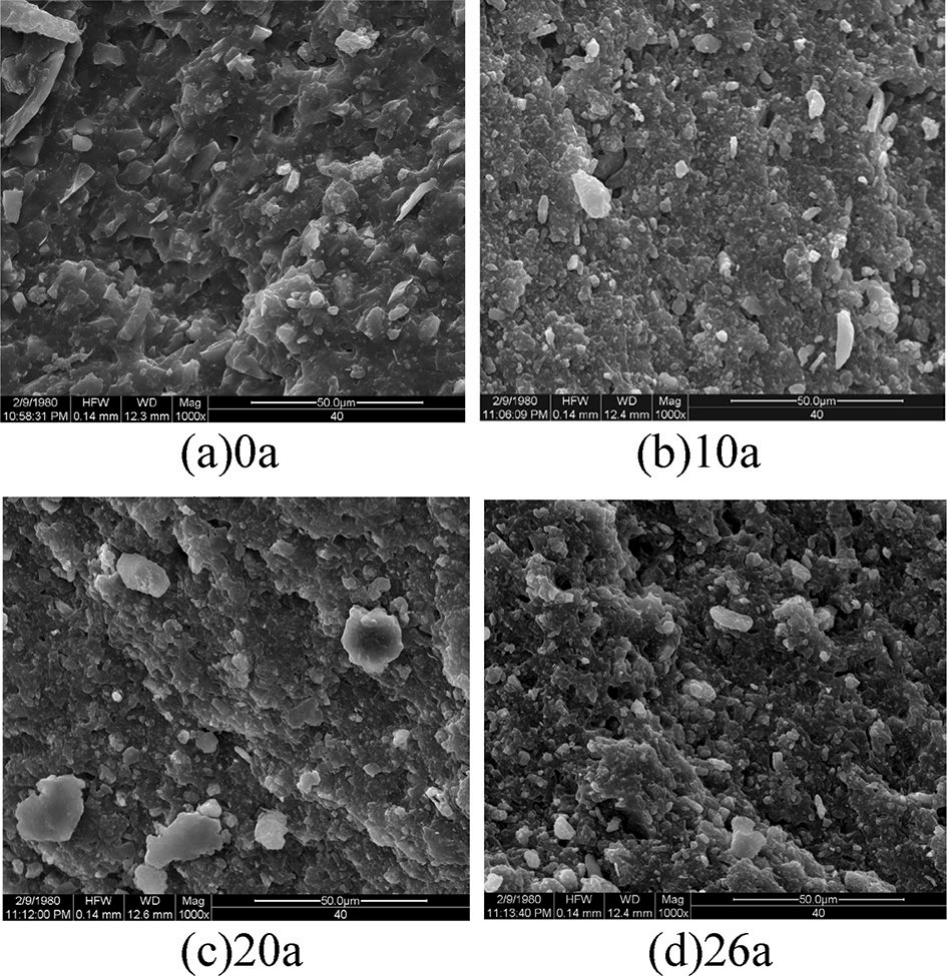
The micro morphology of samples with different operating years.

The micro morphology of the tear section of the shed samples with different operating years is shown in the Fig. 5.

It can be seen that there is a stepped height distribution on the sample surface, which is mainly because the measured surface is a tear section. The white particles on the surface are the fillers in the SIR of the shed. The integrity of the bulk of the virgin sample is good, and the section is smooth. The filler of the shed after operation is precipitated from the bulk and the SIR bulk becomes rougher and fragmented, which will lead to the decline of mechanical properties. The above morphological changes are also one of the characteristics of thermal oxygen aging.

### FTIR

**Figure 6:**
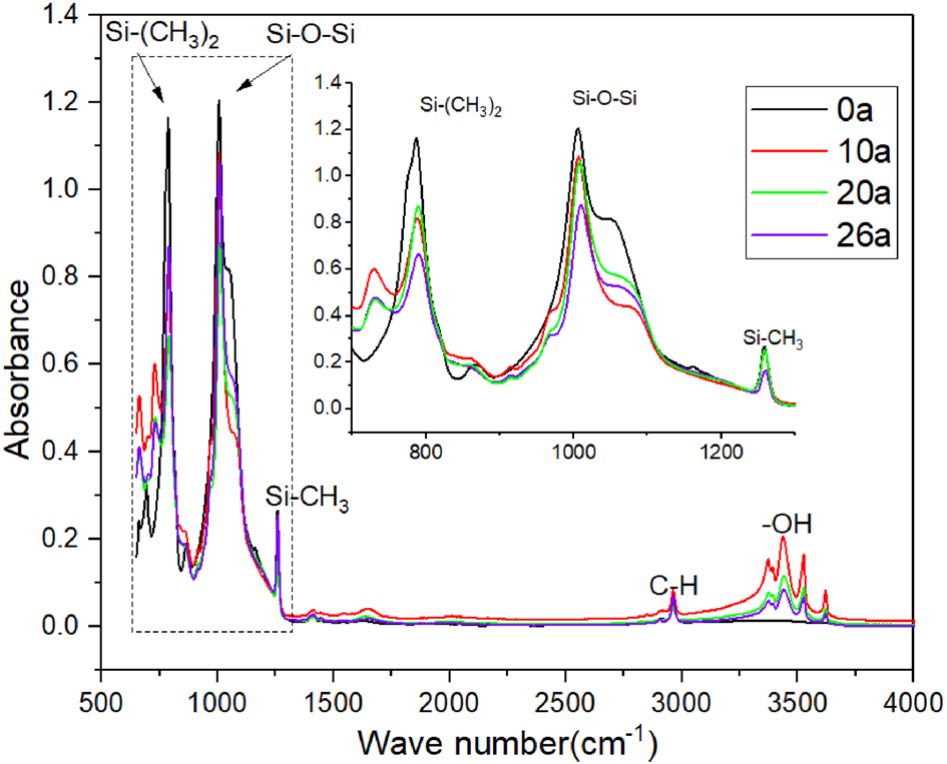
The FTIR spectrum of samples with different operating years.

The infrared spectrum of middle section shed of insulator with different service duration is shown in the Fig. 6.

It can be seen that the sample after operation contains the –OH group, while the virgin sample does not, which indicates that the shed has undergone oxidative hydrolysis reaction to produce hydrophilic group. As the side chain of PDMS, Si–CH_3_ and Si–(CH_3_)_2_ makes SIR hydrophobic macroscopically due to the characteristics of non-polarity and surface orientation. Si–O–Si is the main chain of PDMS, whose content reflects the integrity of molecular chain and is closely related to the aging degree of SIR. The absorption peak heights of characteristic functional groups are shown in the Table 3.

**Table 3:** The height of absorption peak of characteristic functional groups.

Specimen	Si–O–Si	Si-(CH_3_)_2_	–OH
0a	1.204	1.165	0
10a middle	1.085	0.819	0.205
10a high voltage	0.618	0.525	0.198
20a middle	0.877	0.665	0.116
20a high voltage	0.528	0.418	0.123
26a middle	1.064	0.869	0.084
26a high voltage	0.557	0.432	0.146

Compared with the virgin sample, the absorption peak heights of the main chain and side chain of the sample after operation are significantly reduced, which indicates that the chemical bonds are destroyed under the effect of ultraviolet, corona discharge and oxidation. Except for the 20a sample, the hydrophobicity and side chain content of the lightly polluted insulators decreased with the increase of operation duration. The side chain content of 20a sample is the lowest, but the hydrophobicity is good, which is mainly due to the dual effects of heavy pollution on aggravating the destruction of functional groups and adsorbing hydrophobic small molecules.

### XPS

**Figure 7:**
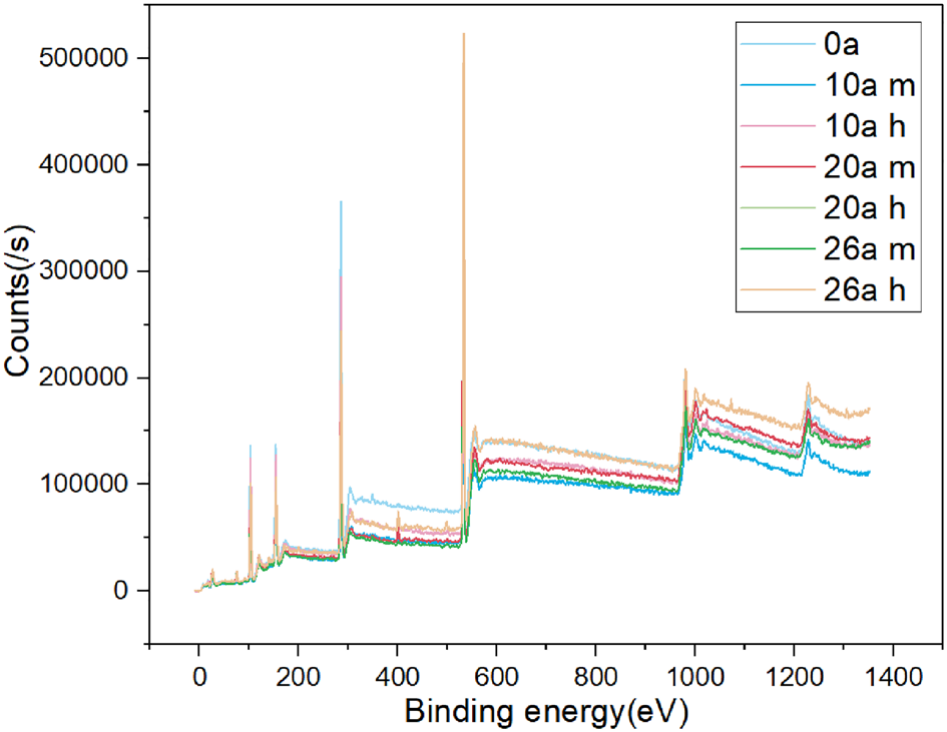
Full scan XPS spectra of samples.

Elements closely related to aging process of the SIR include C, O, Si, Al. The full XPS scan of the samples from 0 to 1300 eV is conducted and the spectrum is shown as Fig. 7.

The content of main elements on the surface of samples with different operating years expressed as atomic concentration is shown in the Table 4.

**Table 4:** The proportion of elements on sample surface.

Specimen	C	O	Al	Si
0a	53.16	25.05	0.55	21.24
10a middle	42.58	31.7	1.92	23.8
10a high voltage	45.55	28.85	2.01	23.6
20a middle	37.99	38.77	3.59	19.66
20a high voltage	30.74	41.85	6.07	21.34
26a middle	38.54	37.09	3.99	20.37
26a high voltage	42.11	35.12	2.79	19.98

As analyzed above, the LMW of SIR will migrate to the pollution layer and then gradually lose during operation. In addition, SIR will undergo thermal oxygen aging and produce CO_2_. These factors lead to a downward trend of C with the service duration. The oxidative hydrolysis reaction on the surface of SIR introduces a large amount of hydroxyl groups, which increases the content of oxygen. During operation, the migration of hydrophobic small molecules causes the gradual loss of Si atoms and the decrease of Si content on the surface of SIR. As the ATH filler gradually precipitates and deposits on the surface, the content of Al increases slightly.

**Figure 8:**
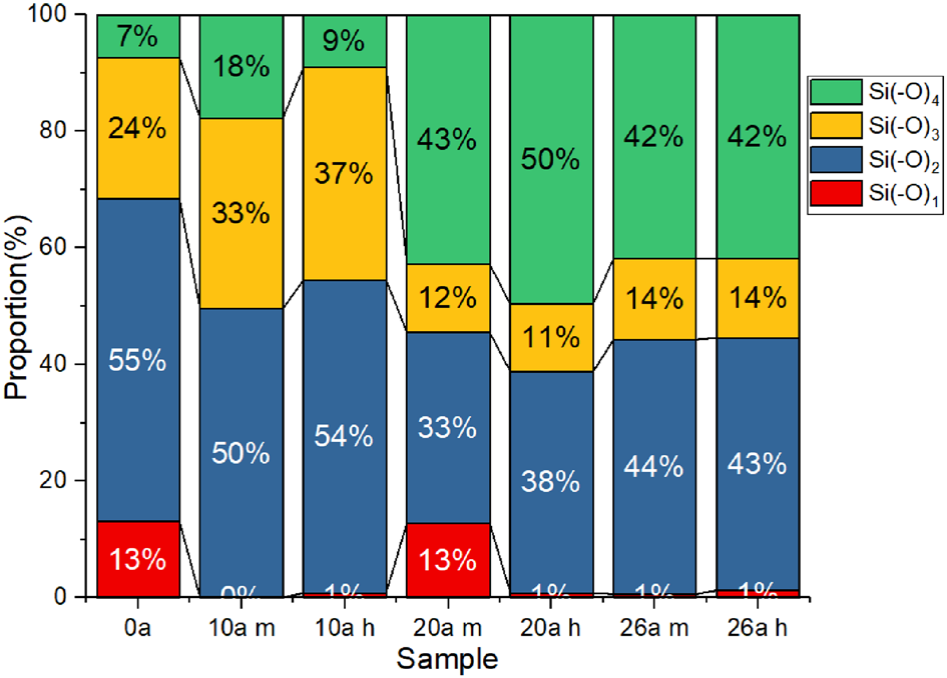
The proportion of silicon atoms with different valence states.

During the aging process of SIR, the oxidation of organic groups and the crosslinking between molecular chains will lead to the change of valence state of silicon atoms. The valence states of Si can be analyzed by fitting and calibrating its XPS spectral peaks. Firstly, the background of the sample spectrum is removed, and the spectral peak position of the sample is corrected by the standard binding energy of C1s (284.8 eV). There are four chemical states of silicon atoms in SIR, namely Si(–O)1, Si(–O)2, Si(–O)3 and Si(–O)4. The Si2p spectra of the samples are fitted with Gaussian-Lorentz curves drawn at the corresponding binding energy of the four states. The integral area of the peaks of the four states can express their relative proportion in the sample. The proportion of silicon atoms with different states of each sample is shown in the Fig. 8.

It can be seen that the Si(–O)1 content in SIR is very low. The proportion of Si(–O)2 in the virgin sample is very high, indicating that the crosslinking degree of virgin sample is relatively low and most silicon atoms exist in the long chain. There are also some silicon atoms of Si(–O)3 structure, which represent silicon atoms linked to oxygen containing groups or silicon atoms at the cross-linking point of molecular chain. Si(–O)4 represents a structure in which silicon atoms are connected with four oxygen atoms, which is equivalent to the inorganic silica like structure. Compared with the virgin sample, the content of Si(–O)2 in 10a sample decreases and the content of Si(–O)3 increases, which is a sign of the crosslinking degree increase and oxidation of organic groups. The content of Si(–O)2 and Si(–O)3 decreases and the content of Si(–O)4 increases significantly in the 20a and 26a samples, which is due to the transformation of Si(–O)3 structure into Si(–O)4 and the precipitation of silica filler. It can be inferred that the crosslinking degree of the shed surface increased significantly, resulting in the increase of macro hardness. The precipitation of silica filler will increase the gap and reduce the internal friction of the shed, resulting in the decrease of tear strength.

### Aging characteristic and mechanism analysis

The static contact angle of shed generally decreases with the increase of service duration. However, the hydrophobicity of the 20a shed with heavy pollution accumulation is better than that of the virgin sample, which is because the pollutants can adsorb LMW, so that the composite insulator has excellent anti-pollution flashover performance. Attention should be paid to the possible flashover caused by the decrease of hydrophobicity after the pollution is removed. The hydrophobicity loss and recovery are well related to the service duration indicating that this characteristic is determined by the aging degree of the internal SIR bulk.

FTIR result displays that the side chains of PDMS molecules of SIR are gradually destroyed during operation. The variation law of the side chain absorption peak height is inconsistent with that of hydrophobicity, indicating that the macro hydrophobicity is not only determined by the integrity of the side chain, but also affected by the pollution adhesion. In addition, the silicon alcohol group produced by oxidative hydrolysis reaction in operation is also the reason for the decrease of hydrophobicity.

Combined with FTIR and XPS, the main chain of SIR will gradually break and rearrange during operation. Si(–O)2 is gradually transformed into Si(–O)3 and Si(–O)4 to form a cross-linked network. Meanwhile, the organic groups are oxidized, and the surface of SIR gradually changes to inorganic structure. As the flexibility of PDMS molecular chain decreases, the hardness of SIR increases. Accordingly, the increase of crosslinking degree of SIR bulk increases the rigidity of the material, so the elastic modulus in DMA also increases.

According to SEM and XPS, under the effect of external environment, the aging degree difference between surface and internal of SIR generates tension, causing the surface to form cracks. Meanwhile, the internal filler will precipitate and lose. After oxygen enters the crack, the thermal oxygen aging intensifies and expands the pores. Therefore, the tear strength of SIR will gradually decrease. The density of SIR first increases with the increase of crosslinking density, and then decreases due to the precipitation of filler.

Affected by multiple external factors, the aging characteristics of the surface of SIR are complex. The variation law between different parameters and service life can be quantitatively analyzed by Pearson coefficient.

The Pearson correlation coefficient of parameters can be calculated by the following formula.

4 $$\rho_{x,y} = \frac{{{\text{cov}} (x,y)}}{{\sigma_{x} \sigma_{y} }},$$

where cov represents the calculation of the covariance of the two sets of parameters and σ represents the standard deviation. The closer the correlation coefficient is to 0, the worse the correlation is.

The correlation coefficient between material and mechanical parameters and operation time is shown in the Table 5.

**Table 5:** The correlation coefficient between material and mechanical parameters and operated years.

Parameters	R
Static contact angle of polluted surface	− 0.183
Static contact angle of cleaned surface	− 0.608
Hydrophobicity loss	− 0.755
Hydrophobic recovery	− 0.887
Hardness	0.978
Tear strength	− 0.744
Density	− 0.600
Storage modulus	0.733
Loss modulus	0.777

It can be seen that the correlation between hydrophobicity and service duration is poor, and the SIR bulk is mainly affected by thermal oxygen aging. Hydrophobicity recoverability, hardness, tear strength and other characteristics determined by the bulk have a strong correlation with the service duration.

It can be seen that the correlation between the static contact angle of the shed surface and the service duration is poor. The main reason is that the pollution degree of each sample is inconsistent, and the adsorption of heavy pollution on hydrophobic small molecules increases the static contact angle of the umbrella shed. The correlation between density and service duration is relatively low, which is mainly because the density is under the dual effects of the increase of crosslinking degree and the precipitation of filler during service. The changes of other parameters are mainly determined by the aging degree of the SIR bulk, so the correlation with the service duration is relatively good.

## Conclusion

The aging characteristics of insulator shed during operation are analyzed mainly from the aspects of material and mechanical parameters.

When the insulator is slightly polluted, the hydrophobicity of the umbrella shed decreases with the increase of service duration. Heavy pollution can adsorb hydrophobic small molecules, so the umbrella shed of heavily polluted insulator has good hydrophobicity.

The hydrophobic loss angle and steady-state recovery angle have a strong negative correlation with the service duration, indicating that the two parameters are less affected by the surface pollution and are mainly determined by the state of the SIR bulk. The aggravation of aging degree of SIR bulk will reduce the wet flashover resistance of insulators.

The FTIR test result reveals that heavy pollution and high electric field strength can aggravate the fracture of main chain and side chain of siloxane. Hydroxyl groups appear in the umbrella shed after operation, indicating that oxidative hydrolysis reaction occurs. The hydrophobic group content of SIR cannot completely determine its apparent hydrophobicity.

XPS results show that the carbon content of SIR decreases and the oxygen content gradually increases, which confirm the occurrence of thermal oxygen aging. The chemical state of silicon atom gradually changes to Si(–O)4, indicating that the PDMS will undergo the chain breaking process first, and then rearrange, resulting in the increase of crosslinking density.

According to SEM, the surface of degraded SIR becomes granular, the pores increase and the filler precipitates, which promotes the aging inside the SIR bulk.

With the increase of service duration, the density of SIR shed shows an increasing trend, but the correlation is not strong, which is mainly because the internal filler is gradually separated out and lost when the crosslinking density of SIR increases.

Due to the increase of crosslinking density, the hardness gradually increases. The increase of micro pores and the precipitation of filler lead to the decrease of tear strength, which may lead to the damage of umbrella shed and the reduction of insulation strength.

The rearrangement degradation of SIR increases the rigidity and internal friction, so the storage modulus and loss modulus gradually increase. The viscoelastic strength of the umbrella shed decreases with the increase of the service duration and the electric field strength.

The changing characteristics of SIR microstructure reveal the changing mechanism of macro parameters of umbrella shed. It can be inferred that the aging of the shed surface is not only determined by thermal oxygen aging, but also strongly affected by external environmental factors, resulting in the complex change law of relevant characteristic parameters. The interior of the shed is mainly affected by thermal oxygen aging, and the relevant characteristic parameters show the correlation with operated years.

## Data Availability

The datasets used and analyzed during the current study available from the corresponding author on reasonable request. The corresponding author of this paper is Lin Mu.
