# Author Correction: Study on material and mechanical characteristics of silicone rubber shed of field-aged 110 kV composite insulators

**DOI:** 10.1038/s41598-023-51046-8

**Published:** 2024-01-10

**Authors:** Lin Mu, Bo Wang, Jinpeng Hao, Ziyi Fang, Yu Wang

**Affiliations:** 1 School of Electrical and Electronic Information, Xihua University, No. 999, Jinzhou Road, Jinniu District, Chengdu, China; 2 State Grid Ningxia Electric Power Company Electric Power Research Institute, No. 716, Huanghe East Road, Yinchuan, China; 3 School of Electrical and Automation, Wuhan University, No. 299, Bayi Road, Wuhan, China

Correction to: *Scientific Reports* 10.1038/s41598-023-35701-8, published online 6 October 2023

The Acknowledgements section in the original version of this Article was incomplete. It now reads:

“Financial support from the Natural Science Foundation of Ningxia Autonomous Region of China (Grant No. 2021AAC03510), and the Sichuan Natural Science Fund, China (Grant/Award Number: 2023NSFSC0295) is acknowledged.”

The original Article has been corrected.

